# Evaluating the moderating role of governance on the relationships between social inclusion, ICT infrastructure, and financial inclusion

**DOI:** 10.1016/j.heliyon.2024.e33711

**Published:** 2024-06-27

**Authors:** Sonia Bibi, Hassan Zada, Tahira Awan, Wing-Keung Wong, Naveed Khan

**Affiliations:** a International Islamic University, Islamabad, (IIUI), Pakistan; b Department of Management Sciences, Shaheed Zulfikar Ali Bhutto Institute of Science and Technology (SZABIST) Islamabad, 44000, Pakistan; c Department of Finance, Fintech & Blockchain Research Center, and Big Data Research Center, Asia University, Taiwan; d Department of Medical Research, China Medical University Hospital, Taiwan; e Department of Economics and Finance, The Hang Seng University of Hong Kong Address: 500, Lioufeng Road, Wufeng, Taichung, Postal code: 41354, Taiwan

**Keywords:** Financial inclusion, Social inclusion, ICT infrastructure, Governance, Principal component analysis, PCSE

## Abstract

In this paper, we examine the Moderating Role of Governance on the Relationships between social inclusion (SI), Information and communication technology infrastructure (ICT), and financial inclusion (FI) in 46 countries representing a global sample span from 2010 to 2020. We collect the data from the IMF's financial access survey and construct a multidimensional FI index. Based on the FI index, we divide the sample into two sub-samples (med-high level and low-level FI countries). For the empirics, we employed panel-corrected standard errors, fully modified ordinary least squares and dynamic ordinary least squares techniques. We find that SI is negatively related to FI. ICT infrastructure positively influences FI. Further, we find that governance with sound ICT infrastructure and socially inclusive communities enhances FI. The findings of sub-samples are similar to the full sample results except for a promoting effect of SI and governance in the case of med-high financially inclusive economies. Moreover, the Interaction term of governance and ICT infrastructure is insignificant in med-high financially inclusive economies and negatively significant in low financially inclusive economies. Our study reports novel findings which have significant implications for policymakers and financial institutions to effectively develop and implement new policies which strengthen the institutional base, develop digital banking infrastructure, enhance SI to boost up FI and ensure sustainable economic growth.

## 1 Introduction

Financial inclusion (FI) is an important building block for financial development. An essential element of economic development is FI. FI has become more affordable, accessible, and easy for individuals all around the world with the development of Information and communication technology infrastructure (ICT) [1,2]. Primarily, FI is the availability, accessibility and usage of competitive and affordable financial services. FI boosts economic growth, financial stability and employment opportunities. Similarly, FI enhances investment and technological innovation. Moreover, it plays a vital role in the successful implementation of government and health services policies [3,4]. Primarily, Information and communication technology infrastructure has led to the development of new financial technologies that have improved access to financial services, including digital wallets, online payment systems, and mobile banking [5]. However, not everyone has access to and capacity to use financial products and services which is the main problem the world is facing these days [6]. Numerous studies have examined the intimate association between industrialization, trade openness, financial development and sustainable economic growth [[7], [8], [9], [10]]. To pursue sustainable economic growth that is compatible with social sustainability and environmental preservation, intensive efforts are needed for the improvement of environmental quality [11]. Similar to this, social inclusion (SI) is an important element for FI. It is another name for equal opportunity to use public resources without any gender discrimination by all the members of society [12]. SI enhances social cohesion and equality, which strengthens national identity and social capital. In a socially inclusive civilization, people access public resources equally and use them freely to their optimum advantage [[12], [13], [14]]. Similarly, it raises the dignity of marginalized sections of society by giving them more sense of belonging and equal opportunities. SI as a development agenda improves the socioeconomic well-being of all society's members [15]. For the promotion of SI, policy commitments have been made in advanced economies. Countries are too busy fulfilling their current emergency needs and the major policy objectives of promoting social and FI are still not yet made [16].

For a state to achieve both the FI and SI is tough because one is achieved at the expense of the other due to strong impedance to social involvement from those who are happy and satisfied from social exclusion as a source of gains [16]. Identifying the FI and SI link might be a possible remedy as it would be helpful to understand why financial system characteristics differ in socially inclusive societies and social integration characteristics differ in financially inclusive countries [17]. In a socially inclusive society, people are more willing to use formal financial services which positively influences the level of FI. Moreover, the regulatory support for the establishment and progress of social entities adds fuel to the fire. Sound regulations promote social progress, according to Ref. [18], which facilitates the use of financial services.

Only willingness to use financial services alone cannot boost FI unless these services are affordable for the people and easily accessible. A sound ICT infrastructure helps in the availability of financial services at affordable prices. As it can reduce costs as well as save time to avoid any inconvenience in accessing financial services. It promotes innovations, which improves performance, according to Refs. [[19], [20], [21]], and reduces poverty as tools which enable the poor to access credit markets, capital markets and a number of financial services [22]. A shift in the role of banks from offering traditional banking services to digital financial services through a wider technological network, start-up and entrepreneurial technology firms. Mobile money (Technological innovation) enhances FI in developing countries as it facilitates financial transactions in the absence of a bank account [23]. ICT infrastructure acts as a bridge to cross the financial, and infrastructural gap by branchless banking facilities to promote FI [24]. It reduces information asymmetry between lenders and borrowers, according to Ref. [25], due to the availability of information at the right time. Similarly, it is the fastest way to enhance FI [26].

As a commercial opportunity, ICT lowers the cost for marginalized sections of society but in many developing countries, it is difficult to replicate and results in financial exclusion [27]. Hence, for efficient distribution of financial products and services, greater access to capital market and financial services, and improved ICT infrastructure are crucial [22]. ICT infrastructure speeds up financial processes resulting in greater financial inclusiveness. The success of ICT infrastructure is associated with appropriate regulatory frameworks [28]. Because for ICT penetration and ICT-led innovations, regulatory frameworks provide support according to Ref. [29], which enhances the effect of ICT infrastructure on financial inclusion. For the coherent performance of financial transactions, governance in the form of a strong rule of law, sound regulatory quality, effective government, political stability and accountability, plays the role of lifeblood in a country [30].

Similarly, good governance acts as a catalyst which speeds up the process of financial inclusion by increasing bank accounts ratio and deposits [30]. While delivering financial products and services governance affects both supply side and demand side determinants of financial inclusion [31]. Regulatory settings acting as the decisive factor can strengthen or weaken their association especially in developing countries [32]. Therefore, it is argued that good governance can support the efficient functioning of financial institutions and give a boost to the level of financial inclusion.

However, governance has been studied as an explanatory variable, according to Ref. [30], and the moderating role of governance in investigating the determinants of financial inclusion is still not examined. The moderation analysis in FI literature is also scarce, according to Ref. [33], and [34], proposed institutional factors as possible moderators of determinants of financial inclusion. 10.13039/100017146FI itself is a premature area due to the lack of required data and little theoretical support [35]. As per directions of [22], other interventions, new ideas, and strategies that promote financial inclusion need to be inspected. Moreover, limited studies investigated the SI-FI link at cross-country and regional levels [19,36], and recommended further testing the SI-FI link to confirm the impact of sustainability policies on financial inclusion across regions, and countries. The association between ICT infrastructure and FI also requires more exploration. So, there is a need to conduct further investigation using a comprehensive set of indicators of FI. Moreover, the direct associations between SI, ICT infrastructure and FI have been studied but the indirect mechanisms are underexplored. To fill these gaps, a research study is needed to evaluate (1) how SI and ICT infrastructure are related to FI? (2) how does governance moderate these links? This study contributes to the existing literature in various ways. First, it adds to the FI literature by highlighting important drivers of FI. Second, this is the first study investigating the moderating role of governance in the SI-FI link and ICT infrastructure-FI link. Exploring such associations helps to understand the complex relationship between socio-economic, technological, and institutional factors and highlights the importance of governance in encouraging more socially inclusive societies and leveraging ICT infrastructure for enhancing financial inclusion. Third, this study contributes to the economic development literature [37], by examining the effect of SI on FI. Fourth, this study is unique because it comprises a comparative analysis of sub-groups having high-med-low level FI. It is helpful to identify drivers of FI in countries possessing med-high FI. It will enable low financially inclusive economies to raise the level of FI. To resolve this issue, we employ the PCSE, FMOLS and DOLS techniques for estimation. We find that SI and ICT infrastructure are significantly related to FI. Furthermore, we report the moderating role of governance in the presence of sound ICT infrastructure and in socially inclusive communities which enhance FI. Finally, our findings are useful for policymakers and financial institutions to effectively develop new policies, improve existing ones by including important factors required to boost FI and implement policies relating to FI to achieve the 2030 targets of sustainable development. Findings are also useful for policymakers in designing inclusive growth strategies. It highlights the need to invest in the creation of more equitable and inclusive societies, ICT infrastructure and governance reforms to maximize desired outcomes Because, strong governance promotes an enabling environment for social cohesion, inclusive growth and sustainable development which boosts up level of financial inclusion.

The remaining part of the paper is arranged in the following order: Section 2 contains a review of the literature. Section 3 covers data and methodology. Section 4 consists of a discussion of findings. Section 5 covers the conclusion and policy recommendations.

### 1.1 Theoretical framework

#### 1.1.1 Institutional theory

Institutional theory highlights the role of institutions and political, social and economic systems. As per the earlier perspective, institutions make policies which regulate institutions. For collective action, institutions provide a regulated platform [38]. The recent view of institutional theory favours the theory of learning as individuals and organizations to understand why and how a specific action is happening in a specific context. For this purpose, attention is given to the way information is gathered and used [39]. One assumption of this theory is: that people have incomplete information. To get more information they interact with institutions. Their recurrent interaction with institutions shapes their behaviours and affects their decisions regarding joining the financial system [36]. This theory relies on a comprehensive set of structures, processes, markets and policies which helps understand the role of governance in attaining high level FI [40]. Research regarding the institutional environment is supported by the institutional theory. The institutional environment is a combination of government governance, legal system, and social and economic setting. It represents a set of factors affecting institutions. The policy environment is a representation of a formal institutional environment and comprises government, educational, legal settings and level of public finance expenditures [41]. A country's political environment and regulatory system affect technological innovations [42] and SI level [43] and people's attitudes towards financial institutions [44].

#### 1.1.2 Collaborative intervention theory of financial inclusion (FI)

According to collaborative intervention theory, FI can be accomplished by collaborative intervention from multiple stakeholders. For the elimination of financial exclusion, a joint effort is required from multiple stakeholders [45]. The range of broader efforts for the delivery of financial services to a wider client base which results in expansion of financial systems, is referred to as FI interventions. Commercial banks, microfinance institutions, financial companies, government programs, village savings and loan associations (VSLAs), mobile network operators, self-help groups, and technology firms are the major service providers. Recently, government, service providers and development funders put a great emphasis on the use of digital means for the delivery of financial services. Technology has gained popularity in East Africa and South Asia, while other regions are still on their way to achieving a peak level in the usage of technology [46]. Technology firms, financial institutions, and telecommunication companies are the collaborative stakeholders, offering digital financial products to promote FI. In a socially inclusive society, people are more willing to use formal financial services which positively influences the level of FI [47]. Similarly, people in a society without SI, do not participate in financial activities.

### 1.2 Literature review

#### 1.2.1 Social inclusion (SI) and financial inclusion (FI)

SI is defined as the process of ensuring the same opportunities which is accessed to all members of society [12,48]. Full participation of all the members of society is ensured through SI policies acting as interventions [47]. The barriers are reduced due to SI policies that allow people to fully participating in society. There are various factors affecting rates of SI such as lack of finances to support social enterprise improvement, the different interpretations of SI, and community enterprises absence [45]. SI's indicators pointed out from the past research work include gender equality, building human resources, public resource utilization equity, social protection, social technology, discrimination and environmental sustainability [6]. According to recent studies, the mainstream SI indicators are social protection, gender equality and environmental sustainability [16]. The advocacy of social protection, gender equality and environmental sustainability are the main causes of social activism. These areas are the main protesting areas against corporations and can induce to modify the behaviour of corporations [17].

In the recent study [44], examined the role of these SI indicators individually as SI policies on FI. He also tested the impact of institutions on FI. His main findings showed a negative impact of SI policies and institutions especially environmental sustainability policies on FI for older people. He found similar results using the SI cluster variable. In another study [45], tested the SI-FI link using correlation analysis and found SI positively correlated with FI for Asian countries, African countries and Middle Eastern countries but failed to find any evidence for European countries. These studies provide contradictory results and there is a need to further test the link to find a clear picture. Similarly [49], also found social trust is a significant and positive determinant of FI. The willingness to use financial services is enhanced with more trust originating from SI. The positive SI-FI link is derived from societal trust and the financialization of society. In a similar vein [50], found a positive effect of social networks on FI of low-income households in Uganda. Similarly [51], showed social progress programs facilitate FI programs and motivate the poor to demand more financial services. Most of the studies favour a positive association between social norms and FI. Therefore, it is hypothesized that:

H1 SI influences FI

#### 1.2.2 Information and communication technology infrastructure (ICT) and financial inclusion

Human life has changed due to the rapid development of technology. A change in the behaviour of the people has been observed due to technological developments in the field of financial services [52]. Therefore, technological innovations have been encouraged and practically implemented in the financial service industry. For this purpose, digital platforms have been developed to facilitate their customers' daily transactions by reducing costs and making the process easy and less time-consuming. Technological applications such as mobile banking and electronic payment systems are popular these days [53]. To offer technological support, companies join their resources for the delivery of financial services. Because, a supportive effect on the financial service's access, has been observed due to technological developments [53,54]. Technological developments in the financial service industry offer innovative channels which increase the rate of participation of unbanked segments of the economy. An effective and affordable technological tool is a mobile phone which promotes FI [27]. Recently, the use of digital technologies was encouraged by the government in China for the enhancement of FI and social stability, as there was a fast evolution of Fintech and increased demand for services [55]. Deployment of technology-enabled models and financial innovations are required for the enhancement of FI. Government practices, policies and business models have been changed through this process of transformation. FI can be increased with the help of technology as it is a remedy to structural and infrastructural issues restricting access to financial services for poor ones [56]. Similar to this, ICT infrastructure in the form of broadband, physical hardware, internet, transmission media, software and internet protocols leads towards sustainable technological progress [22]. To achieve this end, ICT acts as a way to provide banking services to poor ones and the ICT governance framework enhances the quality of these banking services [57].

The effects of ICT have been extensively documented by previous studies [58,59]. According to Ref. [24], geographical diffusion of banking services, mobile money, and internet banking are the results of ICT implementation and are part of strategies relating to FI [60]. reported a significant contribution of physical infrastructure, ICT-based financial products and services boosted the FI levels in India [61]. found ICT infrastructure enabled the poor to access and use financial products and services by reducing cost, and insecurity and boosting public confidence. In another study [62], argued that for the effective delivery of credit to financially excluded ones, ICT can reduce barriers and enhance FI. Similarly, information asymmetry between lenders and borrowers, shrinks with the use of ICT [63], due to the availability of information at the right time. It results in higher bank credit and broader information-sharing platforms, because of which, the level of FI rises [64], and restrictions on financial accessibility are reduced. A recent study [65], documents an unveiled connection between the development of ICT and FI and a combination of both results in sustainable growth. Similarly [26], highlighted that implementation of ICT speeds up the FI process and the economy would grow. Further, it finds that ICT fosters FI and results in the reduction of poverty and inequality. Moreover, ICT infrastructure acts as an instrument that promotes digital finance which is the fastest way of enhancing FI. Similar to this, ICT brings convenience, and low-cost advantage for the financially excluded ones resulting in the enhancement of FI [66]. Therefore, it is hypothesized that:

H2 ICT Infrastructure Positively Influences FI

#### 1.2.3 Governance as moderator

For enhancement of FI, better institutional quality boosts trust in the financial system [63]. The way of interaction and behaviour of economic agents is affected by governance as uncertainty is increased, resources are misallocated and markets become dysfunctional in the presence of a weak institutional framework [67]. While strong institutional environment depicting good governance, rule of law, and no corruption, reduces uncertainty.

Governance affects both supply side and demand side determinants of FI [31]. According to Ref. [68], regulatory setting acts as the decisive factor which may strengthen or weaken the association specifically in developing countries [43]. also argued both SI and digitalization are under the influence of the governance of every set of countries. Similarly, social development is gained through a sound legal and regulatory framework. As suggested by Ref. [45], equal access to financial products and services, and legal protection from exploitation by financial institutions are the complementary benefits attached to sound governance. Regarding the adoption of technology, past studies have examined the role of environmental factors including the institutional environment [69,70]. According to Ref. [28], ICT infrastructure is associated with appropriate regulatory frameworks. [71], documents the significant interaction effect of ICT infrastructure and governance on socio-economic development. It means that ICT infrastructure solely is not responsible for FI, comprehensive and supportive regulation environment is mandatory. For ICT penetration and ICT-led innovations, regulatory frameworks provide support [29]. Similarly [72], confirmed the moderating role of governance on the link between ICT and income inequality. Therefore, it is hypothesized that

H3 Governance strengthens the relationship between SI and FI.

H4 Governance strengthens the relationship between ICT infrastructure and FI

## 2 Data and methodology

### 2.1 Data

We extracted the data from the IMF financial access survey and constructed a multidimensional FI index for 46 countries representing a comprehensive sample over 2010–20. Moreover, data for SI, ICT infrastructure and governance have been extracted from the World Bank, country policy and institutional assessment indicator, ITU, and Worldwide Governance Indicators Database. Further, our analysis began with all 189 countries included in the Financial Access Survey, however, the country policy and institutional assessment indicator database of the World Bank contains data for 83 countries. Most of the countries out of these 83 countries have missing values of either financial inclusion indicators or ICT infrastructure indicators. Further, we removed countries with missing data for FI, SI and ICT infrastructure indicators. Our final sample consists of 46 countries having a complete data set of all variables of interest. A list of countries is provided in Appendix (Refer to Table A2 a.).

### 2.2 Variables

#### 2.2.1 Construction of FI index

Various studies used principal component analysis (PCA) to construct the FI index [73,74]. Following the earlier approach, we compute an index by combining three FI dimensions i.e., availability, accessibility and usage of financial services. The number of ATMs and the number of branches, both per 100,000 adults have been employed as the availability dimension of FI. The number of ATMs and the number of branches, both per 1,000 ${km}^{2}$ have been utilized as the accessibility dimension of FI. Outstanding deposits and outstanding loans by banks, both scaled by GDP have been used as indicators of usage dimension depicting actual use of financial services.

Further, we followed the procedure adopted in past studies for the construction of financial development indices [75], the human development index, and the FI index [76], comprising three steps. The initial step is to get at a common scale ranging from 0 to 1 through normalization. For this purpose, six FI's indicators have been normalized by exploiting data-based normalization.

(1) $$I_{i,t,c}^{n}=\frac{I_{i,t,c}-\mathrm{Min}\left(I_{i}\right)}{\mathrm{Max}\left(I_{i}\right)-\mathrm{Min}\left(I_{i}\right)}$$

In Eq (1) the FI's indicator value i is represented by $I_{i,t,c}$, t symbolizes period, c is for country $\mathrm{Max}\left(I_{i}\right)$ and $\mathrm{Min}\left(I_{i}\right)$ are the maximum and minimum values of indicator i, respectively, over the sample period for all representative countries. Therefore, the deviation of the indicator's value from the given range is represented by the normalized value across the sample, which relates the level of FI indicators of a country to global maximum and minimum levels.

In the second step, weights have been assigned to the normalized indicators of three dimensions with the help of a principal component analysis. We considered only those principal components having eigenvalues greater than 1 for the weight selection. We calculated the Index for each dimension on the basis of assigned weights by taking a weighted average of two related indicators. After calculating the indices for sub-dimensions, again PCA is run to assign the weights to sub-indices. We followed the same weight selection criteria considering only those components having eigenvalues greater than 1. Finally, with the help of calculated indices of sub-dimensions in the first stage PCA and their assigned weights in the second stage PCA, an aggregate FI index has been constructed in the following manner:

(2) Financialinclusionindex=ω1*Availabilityindex+ω2*Accessibilityindex+ω3*Usangeindex

Where in Eq (2) ω represents the weight assigned to a dimensional index. We again used Eq (1) to normalize the calculated FI values. Greater FI is indicated by a higher value of $I_{i,t,c}$ , but within the range of 0 and 1.

Further, we follow [77], to decide the level of FI and categorized the sample under study on the basis of the value of the FI index as

1 0 ≤ FII ≤0.4, indicates low FI
2 0.4 < FII ≤0.6, indicates medium FI
3 0.6 < FII ≤1, indicates high FI

Only Uzbekistan and Honduras countries fell into the category of medium FI and 7 (Armenia, Bangladesh, Bosnia, Cabo Verde, Georgia, Moldova, Mongolia) countries have a value of FI greater than 0.4 for 2010–2011 while greater than 0.6 after 2012 and onwards. India & Mauritania have a value of FI greater than 0.6 from 2014 onward. So, we merged these two categories into one med-high-level FI. The list of countries in the sub-samples is given in Appendix (Refer to Table A2 b.).

#### 2.2.2 SI and ICT infrastructure

We used cluster variables containing the SI and equity policy as a proxy of the level of SI. This cluster variable gauges the quality of the strategies formulated to boost SI in the domains of gender equality, building human resources, social protection, equity of public resource use, labour policies, and institutions for environmental sustainability. The cluster variable is simply the average value of all indicators and its value ranges from 1 to 6.1 indicates low level SI and 6 represents high level SI. The cluster variable is preferred over the individual indicator of SI as it covers multi-indicators of SI in a single value. [16,17], used cluster variables containing the SI and equity policy as a measure of SI. Accordingly, we employed the same measure in our study. To measure ICT Infrastructure, recent studies used a composite index of ICT indicators. For example [65,78], constructed an index of ICT through PCA. Following recent research in the ICT literature, we also constructed a composite ICT infrastructure index using PCA. Indicators used to build this index consist of wireless broadband subscriptions, mobile subscribers, estimated internet users, and fixed broadband subscriptions each per 100 inhabitants. ITU is the source of the extraction of this data.

#### 2.2.3 Governance

We follow past studies such as [30], and [79] to construct a governance index. The variable description is in Appendix (Refer to Table A1.). Various studies used the PCA technique to construct an index [73,74,78]. Therefore, we employed PCA to confirm the selected indicators for index construction are appropriate. Results of PCA are reported in Appendix (Refer to Table A3 a., A3.b.). We used the PCA technique to construct indices in our analysis because of various reasons. First, recent studies adopted this method. Second, PCA is a dimensionality reduction technique. It removes excessive information by generating uncorrelated, interpretable small units called principal components. It provides a remedy for high correlation among series. It is based on the assumption of linearity and uses normalized data for index construction. Third, this technique assigns different weights to different dimensions instead of assigning equal weights to all the dimensions.

### 2.3 Econometric model

Estimation is based on the econometric description given below:

(3) $${FI}_{i,t}=\alpha +\beta_{1}{SI}_{i,t}+\beta_{2}{ICT}_{i,t}+\beta_{3}{CV}_{i,t}+\beta_{4}{GOV}_{i,t}+{\beta_{5}{{SI}_{i,t}\times GOV}_{i,t}+\beta_{6}{{ICT}_{i,t}\times GOV}_{i,t}+c}_{i}+c_{t}+\mu_{i,t}$$

Where in Eq (3)${FI}_{i,t}$ is the index of financial inclusion for country *i* at time *t*, $c_{i}$ and $c_{t}$ are country and time-fixed effects, respectively and ${\;\mu }_{i,t}$ is the error term. SI denotes social inclusion, ICT stands for ICT infrastructure, GOV symbolizes governance index, and CV represents control variables.

### 2.4 Estimation techniques

Following [80,81] we employed a nonparametric covariance matrix estimator proposed by Ref. [82] and panel corrected standard errors (PCSE) technique for the analysis of full sample. We applied these techniques due to the following reasons; 1. The [82] and PCSE techniques are best when the model suffers from heteroscedasticity, cross-sectional dependence and autocorrelation [83]. Results of preliminary tests confirmed that Heteroscedasticity, cross-sectional dependence and autocorrelation were present in our model. 2. These techniques are equally suitable for balanced and unbalanced panels [84]. 3. Our sample consists of large N (46 countries) and small T (11 years). For such samples [82], and PCSE are the most suitable choices.

Traditional panel estimation methods such as pooled OLS, and random and fixed effect estimators are not a wise choice in our case. These models have shortcomings such as, 1. errors are not spherical 2. provide inefficient estimates in the presence of heteroscedasticity, cross-sectional dependence and autocorrelation [83]. Therefore, we didn't apply these models. Moreover, the Feasible Generalized Least Square proposed by Ref. [85] cannot aptly substitute these techniques. Because it is suitable in the case of small N and large T and it underestimates the standard errors in samples with a large number of cross sections and small period [86] which is our case. That is why we used [82] and the PCSE estimator.

We follow [87], to choose Fully-Modified Ordinary Least Squares (FMOLS) and Dynamic Ordinary Least Squares (DOLS) to analyze data in sub-samples. In sub samples, all variables are stationary at first difference. Moreover, cointegration test confirmed presence of long-run association among variables. In the presence of cointegration in the panel series, pooled least square estimates became biased and inconsistent. Panel cointegration techniques (FMOLS and DOLS) resolve this issue. Moreover, our models may suffer from endogeneity and heterogeneity issues because these issues are common in panel data. Both FMOLS and DOLS can overcome the problem of autocorrelation, endogeneity [88], heterogeneity and heteroskedasticity, where differences in the variance of residual terms is the cause of heteroskedasticity. These techniques can handle the cross-sectional dependence too. As compare to GMM, these techniques are more reliable because long run estimates produced by GMM are spurious. GMM doesn't consider cross-sectional dependence and cointegration [89]. These techniques are better in estimation as compare to mean group estimators because according to Ref. [90], mean group estimators are seriously biased when N is large and T is small which is our scenario (T = 11, N = 46).

## 3 Empirical results and findings

### 3.1 Descriptive statistics

Table 1a shows the summary statistics of the variables. The mean value of commercial bank branches per one hundred thousand persons is greater than the mean value reported by Ref. [91]. Similarly, the mean value of all the other indicators of FI indicators is less than the average values reported by them. It means that our sample countries are less financially included as compared to South Asian and South African countries. The mean value of outstanding loans and deposits is less than reported by Ref. [92]. It means the level of use of financial services in our sample countries is low. There is a need for more investment in constructing more bank branches and ATMs per 1000 km square. It will increase access to marginalized sections of society and improve the usage of financial services.

**Table 1a:** Descriptive Statistics (Full sample).

Variable	Obs	Mean	SD	Min	Max
BB	506	11.600	14.040	0.363	71.229
ATM	506	15.890	18.004	0.090	106.882
NBB	506	8.026	13.483	0.02	82.43
NATM	506	11.186	16.748	0.02	97.59
OD	506	38.600	32.899	3.672	243.788
OL	506	31.559	29.945	1.753	222.171
FB	506	2.564	4.796	0.0004	25.814
AM	506	26.372	24.638	0.002	124.724
MC	506	81.633	31.024	8.564	149.543
IU	505	22.207	18.205	0.83	76.507
CC	506	−0.701	0.522	−1.645	0.970
GE	506	−0.676	0.434	−1.608	0.809
PS	506	−0.624	0.750	−2.810	0.887
Reg	506	−0.574	0.453	−2.080	1.038
Law	506	−0.685	0.475	−1.922	0.662
VA	506	−0.551	0.643	−2.124	0.974
FI	506	0.291	0.253	0	1
SI	506	3.482	0.385	1.8	4.3
ICT	506	0.552	0.357	0.008	1.767
GOV	506	1.114	0.375	0.212	2.142
ADR (%)	506	73.061	18.813	39.125	106.624
INF (%)	506	8.018	30.562	−26.296	604.945
UPOP (%)	506	39.909	16.050	10.642	78.062
POP (Million)	506	55.943	194.177	0.521	1396.387

Note: In Table 1a, Obs shows the observation, while SD shows the standard deviation and min, and max shows the minimum and maximum value. A list of Abbreviations is in Appendix (Table A4).

The mean values of ICT indicators are greater and the mean values of governance indicators are less than the mean values reported by Ref. [93]. Especially, the mean value of control of corruption and political stability. It indicates a better condition of ICT infrastructure and poor governance especially high corruption and more political instability exists in our sample countries as compared to African countries. It means there is a dire need to strengthen the regulatory framework by reducing corruption and political instability in these countries.

FI index has a low standard deviation as compared to other explanatory variables (SI and ICT infrastructure) and moderating variables (Governance). It means less variations exist in the level of FI across our sample countries. The mean value of SI is higher than the reported mean values in the study [16]. It means our sample countries are more socially inclusive. Age dependency ratio, inflation, urban population and market size have high standard deviations which means these variables vary greatly across countries.

Table 1b reports descriptive statistics of sub-samples i.e., the med-high- and low-level FI countries. The mean values of FI indicators, indicators of ICT infrastructure and governance are higher in the med-high financially inclusive sample than in low-level financially inclusive ones. It means countries in med-high level FI have better ICT infrastructure and regulatory framework. FI, ICT infrastructure and governance vary greatly in med-high level financially inclusive countries as compared to less financially inclusive countries. SI shows less deviation from the average value in the countries with med-high level FI as compared to the countries which are not financially inclusive. It means these countries show a roundabout similar level of SI. The age dependency ratio, inflation, urban population and market size show greater variation in less financially inclusive economies

**Table 1b:** Descriptive statistics (med-high FI and low FI).

	Med-High FI					Low FI				
Variable	Obs	Mean	SD	Min	Max	Obs	Mean	SD	Min	Max
BB	132	26.503	17.798	3.104	71.229	374	6.340	7.021	0.363	68.806
ATM	132	35.260	23.313	2.113	106.882	374	9.054	8.266	0.090	42.307
NBB	132	21.922	19.558	0.09	82.43	374	3.122	4.399	0.02	31.13
NATM	132	30.871	22.070	0.09	97.59	374	4.239	4.814	0.02	28.64
OD	132	64.559	50.235	13.361	243.788	374	29.438	16.013	3.672	117.137
OL	132	61.209	41.603	14.371	222.171	374	21.094	13.610	1.753	119.716
FB	132	7.594	6.901	0.011	25.814	374	0.789	1.518	0.0004	11.916
AM	132	39.039	27.082	0.030	111.505	374	21.901	22.078	0.002	124.724
MC	132	102.524	23.689	45.773	149.227	374	74.259	29.951	8.564	149.543
IU	132	38.144	20.473	3.7	76.507	373	16.567	13.395	0.83	72
CC	132	−0.446	0.590	−1.387	0.970	374	−0.790	0.465	−1.645	0.776
GE	132	−0.333	0.434	−1.043	0.809	374	−0.797	0.3645	−1.608	0.308
PS	132	−0.315	0.612	−1.627	0.887	374	−0.734	0.765	−2.810	0.678
Reg	132	−0.305	0.567	−1.729	1.038	374	−0.669	0.360	−2.080	0.185
Law	132	−0.388	0.480	−1.452	0.662	374	−0.790	0.428	−1.922	0.154
VA	132	−0.346	0.760	−2.124	0.974	374	−0.623	0.580	−1.816	0.596
FI	132	0.669	0.152	0.4	1	374	0.154	0.088	0	0.392
SI	132	3.788	0.244	3.2	4.3	374	3.374	0.368	1.8	4.3
ICT	132	0.881	0.397	0.151	1.767	374	0.436	0.255	0.008	1.497
GOV	132	1.357	0.399	0.541	2.142	374	1.028	0.326	0.212	1.729
ADR (%)	132	53.042	12.246	39.125	91.622	374	80.127	15.318	52.970	106.624
INF (%)	132	6.667	8.389	−11.876	48.581	374	8.494	35.199	−26.296	604.945
UPOP (%)	132	50.074	11.903	30.417	68.657	374	36.321	15.797	10.642	78.062
POP (Million)	132	136.03	362.05	0.521	1396.39	374	27.673	44.051	0.744	227.196

Note: Refer to Table 1a.

### 3.2 Correlation matrix

Table 2 reports the results of the correlation analysis. ICT infrastructure, SI, governance, market size and urban population are strongly and constructively linked with FI. Age dependency ratio and inflation are negatively associated but age dependency ratio has a strong correlation.

**Table 2:** Correlation matrix.

Full Sample								
	FI	SI	ICT	GOV	ADR	INF	POP	UPOP
FI	1							
SI	0.547969	1						
ICT	0.697124	0.517373	1					
GOV	0.475905	0.667285	0.450648	1				
ADR	−0.74682	−0.5354	−0.66951	−0.3967	1			
INF	−0.06005	0.059155	−0.00465	−0.10509	0.025013	1		
POP	0.251723	0.054523	−0.05966	0.112093	−0.15099	−0.0099	1	
UPOP	0.408988	0.114196	0.419257	0.257248	−0.37475	−0.0337	−0.08493	1
Med-High FI								
Variable	FI	SI	ICT	GOV	ADR	INF	UPOP	POP
FI	1							
SI	0.229354	1						
ICT	0.487465	0.535389	1					
GOV	0.55713	0.565651	0.416181	1				
ADR	−0.28646	−0.52757	−0.41843	−0.28443	1			
INF	−0.34995	−0.08642	−0.23709	−0.35978	−0.0681	1		
UPOP	0.048407	0.234915	0.264747	0.389829	0.064296	−0.11506	1	
POP	−0.09146	−0.28353	−0.38318	−0.38224	−0.06784	0.170933	−0.79151	1
Low FI								
	FI	ICT	SI	GOV	ADR	INF	UPOP	POP
FI	1							
ICT	0.397497	1						
SI	0.58288	0.317559	1					
GOV	0.166594	0.63071	0.242496	1				
ADR	−0.6895	−0.31329	−0.56653	−0.19456	1			
INF	−0.06273	0.092998	0.04686	−0.0916	0.01667	1		
UPOP	0.263534	−0.13341	0.299908	0.050975	−0.2441	−0.02117	1	
POP	−0.01097	−0.0176	−0.13254	−0.18896	0.451396	0.044875	−0.09744	1

In the low financially inclusive economies, SI, ICT infrastructure, age dependency ratio and urbanization show a strong correlation with FI as compared to med-high financially inclusive countries. Governance is strongly associated with FI in med-high-level financially inclusive societies. Inflation and market size are strongly adversely connected with FI in countries having med-high level FI. Correlation among explanatory variables in the full sample as well as in sub-samples does not exceed 0.8. It means models are free from multicollinearity.

### 3.3 Cross-section dependence test

In the heterogeneous panel, the cross-section dependence is a common issue and must be examined [94]. Therefore, it is necessary to resolve this issue because it can lead to weakening the efficiency of the panel dataset, size distortion, bias stationarity, as well as spurious results [95]. We employed two different tests of cross-section dependence i.e. Pesaran CD and Pesaran LM test on the following grounds i.e., 1. Breusch–Pagan LM test does not work well with short span panel (N > T) (Pesaran, 2004) and hence is not suitable in our case. 2. Recent studies such as [95], applied these tests. Table 3 reports the outcomes of cross section dependence. As stated by the results, variables are significant at a 1 % level of significance. Hence, the null hypothesis of cross section independence is rejected. These results confirmed the existence of cross section dependence in full as well as in sub-panels.

**Table 3:** Cross-section dependence test.

Pesaran CD						
Variable	Full Sample		Med-High FI		Low FI	
	Stat	Prob	Stat	Prob	Stat	Prob
FI	63.118	0.000***	17.005	0.000***	47.382	0.000***
SI	0.1715	0.863	−0.046	0.9628	2.1346	0.032**
ICT	97.188	0.000***	25.066	0.000***	70.964	0.000***
GOV	3.244	0.001***	6.129	0.000***	0.3229	0.7467
ADR	32.482	0.000***	−0.598	0.5497	50.243	0.000***
INF	20.78	0.000***	9.290	0.000***	13.318	0.000***
UPOP	79.539	0.000***	11.774	0.000***	67.409	0.000***
POP	73.069	0.000***	1.635	0.1018	77.696	0.000***
Pesaran scaled LM						
	Full Sample		Med-High FI		Low FI	
FI	116.833	0.000***	35.544	0.000***	83.979	0.000***
SI	28.287	0.000***	4.448	0.000***	24.498	0.000***
ICT	185.458	0.000***	48.279	0.000***	133.845	0.000***
GOV	60.559	0.000***	12.782	0.000***	45.428	0.000***
ADR	168.897	0.000***	40.932	0.000***	126.204	0.000***
INF	15.352	0.000***	9.3978	0.000***	10.575	0.000***
UPOP	196.533	0.000***	39.401	0.000***	154.205	0.000***
POP	214.452	0.000***	48.419	0.000***	162.573	0.000***

Note: Significance ‘***’ = 0.01 ‘**’ = 0.05 ‘*’ = 0.1.

### 3.4 Panel unit root test

Cross section dependence lowers the predictive power of first-generation panel unit root test and results in size distortions [96]. Following [97], we applied CADF test proposed by Ref. [98]. Table 4 reports the results of CADF test. In full sample, all the variables are significant at 1 % level of significance. It means all the variables are integrated of order I (0) in full sample. In sample of countries having med-high level FI and low-level FI, first-differences of variables are significant at 1 % and 5 % level of significance. It means all the variables in sub-samples are integrated of order I (1). This fact led to the use of different techniques to analyze data in case of full sample and sub-samples.

**Table 4:** Panel unit root test.

Full Sample			Med-High FI				Low FI			
Var	I (0)		I (0)		I (1)		I (0)		I (1)	
	Stat	Prob	Stat	Prob	Stat	Prob	Stat	Prob	Stat	Prob
FI	−2.769	0.001***	−2.018	0.18	−2.427	0.028**	−2.764	0.004***	−3.217	0.000***
SI	−2.205	0.001***	−2.195	0.07*	−3	0.000***	−2.364	0.000***	−3.156	0.000***
ICT	−3.771	0.000***	−1.267	0.999	−3.417	0.005***	−2.565	0.055*	−6.237	0.000***
GOV	−2.808	0.000***	−2.495	0.008***	−2.687	0.005***	−2.62	0.029**	−3.608	0.000***
ADR	−2.865	0.000***	−2.442	0.285	−3.297	0.011**	−4.84	0.000***	−3.71	0.000***
INF	−2.934	0.000***	−3.603	0.000***	−3.974	0.000***	−2.577	0.048**	−3.101	0.001***
UPOP	−2.742	0.002***	−2.589	0.152	−5.784	0.000***	−8.16	0.000***	−7.13	0.000***
POP	−2.1	0.009***	−2.267	0.491	−3.044	0.041**	−1.356	1.000	−2.755	0.038**

Note: Refer to Table 3.

### 3.5 Heteroskedasticity and serial correlation test

Further, we estimated the Modified Wald test for group wise heteroskedasticity and the Wooldridge autocorrelation test as shown in Table 5. The results confirmed that our models are plagued with both heteroskedasticity and autocorrelation.

**Table 5:** Heteroskedasticity and serial correlation test.

Modified Wald heteroskedasticity test					
Full Sample		Med-high FI		Low FI	
Stat	Prob	Stat	Prob	Stat	Prob
5570.5	0000***	123.42	0000***	8407.76	0000***

Wooldridge autocorrelation test					
Full Sample		Med-high FI		Low FI	
Stat	Prob	Stat	Prob	Stat	Prob
77.657	0000***	45.429	0000***	55.91	0000***

Note*:* Refer to Table 3.

### 3.6 Driscoll–kraay standard errors (DKSE) & panel-corrected standard errors (PCSE)

Table 6 presents the results of DKSE and PCSE. Where SI is negatively related to FI. It means that countries with high SI people don't avail the services of formal financial service providers. Instead, due to social cohesion they use informal sources for financial help, which results in a decrease in FI. One possible reason could be poorly designed and implemented social policies for marginalized sections of society [35]. Similarly, low-income individuals in these countries remain deprived of access to financial services either voluntarily or banks deny to provide services to such individuals. Hence, poor social policies fail to promote FI. A recent study confirmed that desired outcomes cannot be achieved through poorly designed and implemented social policies [99]. Similarly [36], documents a significant and negative impact of SI policies on FI in the case of older low-income populations. According to collaborative intervention theory, FI is an outcome of joint efforts from multiple stakeholders [45]. In socially inclusive societies, due to more social cohesion and poor policies, the efforts from these multiple stakeholders such as financial institutions fail to bring desired outcomes. People in socially inclusive societies don't avail the services of formal financial service providers and use informal sources for financial help which results in a decrease in financial inclusion.

**Table 6:** Driscoll–kraay standard errors & panel-corrected standard errors results.

Var	Driscoll–Kraay Standard Errors		Panel-Corrected Standard Errors	
	Coef.	Prob	Coef.	Prob
SI	−0.1055 (0.0230)	0.0010***	−0.0503 (0.0221)	0.0230**
ICT	0.2340 (0.0712)	0.0080***	0.0335 (0.0558)	0.5480
GOV	−0.2753 (0.0480)	0.0000***	−0.1681 (0.0840)	0.0460**
SIGOV	0.1152 (0.0142)	0.0000***	0.0674 (0.0257)	0.0090***
ICTGOV	−0.0265 (0.0416)	0.5380	0.0983 (0.0392)	0.0120**
ADR	−0.0029 (0.0007)	0.0030***	−0.0068 (0.0007)	0.0000***
INF	−0.0007 (0.0005)	0.2280	−0.0002 (0.0004)	0.0000***
UPOP	0.0127 (0.0015)	0.0000***	0.0028 (0.0007)	0.0000***
POP	−0.2622 (0.0652)	0.0020***	0.0270 (0.0091)	0.0030***
Con	4.4152 (1.1725)	0.0040***	0.2586 (0.1638)	0.1140
R-squared	0.5435		0.8125	

Note: Refer to Table 3, Standard errors are shown in parentheses.

The coefficient of ICT infrastructure is significant and positive meaning that sound ICT infrastructure directly influences and promotes the level of FI. Sound ICT infrastructure ensures the immediate supply of credit information which reduces information asymmetries and the cost of financial transactions [27,100]. It reduces barriers to financial access and enables the poor sector of society to access financial services and use them [62]. Findings of [64], and [27], also confirmed that the financial system is expended through transparent contract enforcement and timely supply of financial information. Previous studies also found a positive association between ICT and FI [100]. Similarly, ICT infrastructure is insignificant and doesn't directly influence FI. Such results are harmonized with the findings of [101].

Collaborative intervention theory provides support for these results. Technology firms, and financial institutions acting as collaborative stakeholders build a sound ICT infrastructure with a joint effort which increases FI. Moreover, these collaborative stakeholders offer digital financial products to promote FI [16]. Governance is also negatively related to FI. This means that excessive regulatory restrictions result in a decrease in FI [102]. The direct effect of governance is detrimental to FI in low and middle-income countries. [103], confirmed the adverse effect of regulations on FI. The rationale behind the adverse effect of governance on FI is a tight regulatory environment which creates difficulties for new account openers, increases compliance costs, and develops a risk averse behaviour among financial institutions [[104], [105], [106]]. It results in a decrease in aggregate lending and an increase in capital rationing. Banks showing a risk averse behaviour became more cautious in lending and increased lending rates and service charges. In such a situation, low-earners find it more difficult to access financial services. Findings of [107], also support the adverse effect of governance on 10.13039/100017146FI. Our findings are contradictory to the study of [79].

The interaction term of SI and governance is significant. It means that the combined effect of SI and good governance results in an increase in FI. A possible justification for this finding is connected with the ability of regulatory institutions to limit or promote specific actions or behaviour. Regulations also influence social behaviour [108], and increase SI which raises the level of FI [43]. Moreover, a sound regulatory environment ensures that everyone including the poor gains from the implementation of public policies including social welfare policies. Which simultaneously increases the trust and willingness of the poor to engage in financial activities [109]. Financial institutions also support such social policies and programs by delivering affordable financial services to the low-earners. Good governance also ensures fair distribution of economic and financial resources among all society members [92]. This step reduces income inequality, enhances the welfare of all people, especially the poor and promotes FI.

Similarly, the interaction term of ICT and governance is insignificant. According to the findings of PCSE, the combined effect of ICT and governance is also significant and positive which means that ICT and governance together promote FI. A good regulatory framework provides support to build up sound ICT infrastructure [29]. ICT infrastructure enables the poor to use and access financial services by reducing cost, and insecurity and boosting public confidence [62], which increases FI. ICT and governance jointly contribute to the advancement of democracy and promote fair distribution of income [110]. Similarly [111], documents a positive influence of interaction terms of ICT and governance on income distribution. Further, it finds that fair distribution of resources encourages the poor to use and access financial services. These results prove the moderating role of governance.

These results verifying the moderating role of governance are supported by institutional theory. According to this theory, institutions are a comprehensive set of structures, processes, markets and policies which highlight the role of governance in attaining high level FI [40]. People possess incomplete information. To get more information they interact with institutions. Their recurrent interaction with institutions shapes their behaviours and affects their decisions regarding joining the financial system [44]. Social norms and regulations are part of institutions which are helpful to raise the level of social inclusion [43]. Moreover, a sound regulatory environment ensures the successful implementation of welfare policies which boost trust in financial institutions according to Ref. [109], and raise the level of financial participation of marginalized sections of society. A country's political environment and regulatory system affect technological innovations [42], and their adoption by financial institutions. A good regulatory framework encourages financial institutions to build up sound ICT infrastructure [29]. Governance and ICT infrastructure jointly promote fair distribution of income [110], reduce cost, and insecurity and boost public confidence [62], which quickly raise the level of financial inclusiveness of the poor, women and other financially excluded sections of society.

Age dependency ratio and inflation are negatively related to FI while urbanization and market size are related positively to FI. A lower age dependency ratio, Urbanization and bigger market size mean greater people demanding financial services and an increase in FI [74]. Low inflation also encourages people to save more and demand more financial services [56].

### 3.7 Analysis of sub-samples

#### 3.7.1 Kao (1999) panel Co-integration

Different panel cointegration tests have been developed by the researchers [112,113]. In the case of small-time dimension T, the Kao panel co-integration test produces more reliable results compared to the Pedroni co-integration test [114]. We performed [113] panel co-integration with the demean option as in Table 7. Demean option removes cross-section means from the series to alleviate the impact of cross-sectional correlation [114]. The null hypothesis of the absence of cointegration is rejected at a 1 % significance level in both sub-samples. Results confirmed there is long-term relationship among the variables.

**Table 7:** Kao (1999) panel co-integration.

Med-HFI	
Statistic	Prob.
−3.39064	0.0003***

LFI	
Statistic	Prob.
−3.69182	0.0001***

Note: Refer to Table 3.

#### 3.7.3 Panel cointegration regression

Table 8 reports the results of FMOLS and DOLS in both sub-samples. SI is significant in both samples but with opposite signs. SI is positively linked with FI in countries possessing med-high levels of FI. It means that in these countries high SI level promotes FI. [115], found that increased social cohesion generates social networks which raise the level of FI. SI enhances knowledge of the people and their prolific capabilities which enable them to avoid poverty and to be more inclusive in the economy [116]. Eventually, people are more financially inclusive. In another study [16], also found a positive association between SI and FI.

**Table 8:** Panel cointegration regression.

Med-High FI				
Variable	FMOLS		DOLS	
	Coefficient	Prob.	Coefficient	Prob.
SI	0.2082 (0.0090)	0.0000***	0.1456 (0.2269)	0.5224
ICT	0.1690 (0.0041)	0.0000***	0.1541 (0.0735)	0.0384**
GOV	0.1858 (0.0344)	0.0000***	0.1337 (0.6017)	0.8245
SIGOV	0.0714 (0.0075)	0.0000***	0.0879 (0.1417)	0.5361
ICTGOV	−0.0039 (0.0032)	0.2224	0.0060 (0.0472)	0.8985
ADR	−0.0102 (0.0003)	0.0000***	−0.0109 (0.0039)	0.0068***
INF	−0.0029 (0.0009)	0.0000***	−0.0012 (0.0008)	0.1668
UPOP	0.0160 (0.0004)	0.0000***	0.0159 (0.0069)	0.0233**
POP	−0.3325 (0.0298)	0.0000***	−0.2786 (0.2562)	0.2792
Adj R-squared	0.8878		0.8643	

Low FI				
Variable	FMOLS		DOLS	
	Coefficient	Prob.	Coefficient	Prob.
SI	−0.0693 (0.0025)	0.0000***	−0.0648 (0.0149)	0.0000***
ICT	0.3174 (0.0035)	0.0000***	0.2925 (0.0496)	0.0000***
GOV	−0.0552 (0.0084)	0.0000***	−0.0658 (0.0605)	0.2774
SIGOV	0.0415 (0.0024)	0.0000***	0.0445 (0.0167)	0.0082***
ICTGOV	−0.1301 (0.0027)	0.0000***	−0.1209 (0.0330)	0.0003***
ADR	0.0007 (0.0004)	0.0000***	0.0011 (0.0006)	0.0974*
INFLATION	−0.0006 (0.0005)	0.0000***	−0.0007 (0.0002)	0.001***
UPOP	0.0004 (0.0001)	0.0008***	0.0005 (0.0020)	0.9953
POP	−0.0485 (0.0023)	0.0000***	−0.0126 (0.0375)	0.7361
Adj R-squared	0.9487		0.9374	

Note: Refer to Table 6.

In low financially inclusive countries, SI is negatively related to FI. It means in these countries high SI level fails to raise the level of FI. These countries are low-income countries too and their low incomes deprive them of to access financial services even in the presence of high SI [35,44,93,116]. ICT infrastructure is significant and positive in both samples. Similarly, sound ICT infrastructure can raise the level of FI irrespective of the level of FI already possessed by the country [32,103]. Similarly, governance is also significant in both sub-samples but shows the opposite direction. Governance is associated positively with FI in moderate to highly financially inclusive economies. It means that in these economies, sound regulations lead to a high FI level. In low financially inclusive countries, governance is associated negatively with FI. It means that in these countries governance results in a decrease in FI. Therefore, various factors could be responsible for it from low income to corruption, ineffective government, political instability and poor accountability [95,102].

The SI and governance jointly produce a positive and significant impact on FI in both sub-samples. High SI with sound governance results in an increase in FI. People feel dignified and secure in socially inclusive and well-regulated economies which increases the trust and willingness of the poor to engage in financial activities [43,109,117]. ICT infrastructure and governance fail to produce any significant impact in med-high financially inclusive economies. In low financially inclusive economies, the combined effect of ICT governance is negative. Despite technological advancement, low income, weak governance and regulatory inefficiencies undermine the efforts to promote FI [118]. Age dependency ratio, inflation and market size are negatively associated with FI. urbanization is positively associated with FI in med-high financially inclusive economies. It means that a higher age dependency ratio, inflation and bigger market size weaken the effects of strategies to raise the level of FI. Because it raises financial stress levels in the economy and reduces demand for financial services [76,[119], [120], [121], [122]].

## 4 Conclusions

In the recent past, the empirical literature on sustainable growth and its intimate relationship with FI has drawn the attention of many scholars around the world. However, a system that presents new prospects to close the wealth disparities in the developing world has evolved with the introduction of ICT and its connection to financial platforms. To achieve this objective, we examine the interlinkages between SI, ICT infrastructure, governance and FI in 46 countries representing a global sample span from 2010 to 2020. Therefore, we first generated the FI index and then categorized countries as high-medium (0.4 < FII ≤1) and low financially inclusive sub-samples (0 < FII ≤0.4). Our model suffers from heteroskedasticity, autocorrelation and cross section dependence. In this regard following recent studies, we applied Driscoll and Kraay's estimator and PCSE techniques for analyzing full samples. To analyze sub-samples, we employed FMOLS and DOLS techniques. Our findings revealed that the influence of SI is negative on FI while the coefficient of ICT infrastructure is positive. It means that SI demotes FI while ICT infrastructure has a promoting direct effect on FI. Interaction terms of both the explanatory variables i.e. SI and ICT infrastructure with governance are positively associated with FI. These findings confirm the moderating role of governance. Governance as a moderator strengthens these links among SI, ICT infrastructure and FI.

Findings slightly differ in analyzing sub-samples. SI and governance are positive and significant in the case of med-high financially inclusive economies. The interaction term of governance and ICT infrastructure are insignificant in med-high financially inclusive economies and negatively significant in low financially inclusive economies. Low income, low technological advancement, corruption and regulatory inefficiencies create difficulties in the integration of ICT and good governance. It results in a decrease in FI. The combination of high SI and good governance is significant and positive in both sub-samples. High SI and good governance jointly promote FI. These findings support the role of governance as a moderator.

Findings recommend a combination of a socially inclusive community, sound ICT infrastructure, and efficient governance framework is required to boost up level of FI. Policymakers should consider these elements. Policy makers should design strategies to control corruption and malpractices which will result in effective governance and high institutional quality. Policymakers should frame proper FI regulations to attain strategic development goals through higher FI. Cost-efficient technology innovations resolving consumer problems should be a part of the strategy. Moreover, Digital literacy programs should be designed and implemented properly to enable people to effectively use ICT tools. Digital government services should be expended to raise citizen engagement, citizen participation in decision making and a more inclusive society. A proper monitoring and evaluation framework should be established by the government to ensure the successful implementation of social welfare policies and financial inclusion initiatives. Special attention should be given to improving the enabling environment for FI, particularly in low income countries. Similarly, financial institutions should build up sound ICT infrastructure, enact robust data privacy and security regulations and promote digital payment systems.

Our sample consists of 46 low- and middle-income countries. It is the major limitation of this study. It lowers the generalizability of our findings. The period for our analysis is also limited (2010–2020). Future scholars should include a wider set of countries with longer periods. Availability of data is limited and our estimation may suffer from potential measurement biases which affects the generalizability of findings. Future research should use alternative measures and try to deal well with these limitations and potential measurement biases. Data for SI is not available for the majority of the countries. Hence, alternative proxies of SI should be applied in future studies. Our analysis is purely quantitative and based on secondary data. Future studies should perform qualitative analysis using primary data. Our analysis used six indicators and three dimensions of FI. Future studies should include other dimensions and indicators of FI and perform a comparative analysis among regions, income groups and countries to derive useful insights.

Further investigation is required to explore the relationship between SI and FI with some other possible moderators such as culture, demographic characteristics and financial freedom. Future studies should investigate the influence of ICT infrastructure on environmental sustainability and social equality. Future studies can also observe the impact of new technological innovations on the outcomes of FI. The dark side of FI is not discussed in the literature. There is a need to recognize the risks connected with excessive FI and ways to mitigate these risks. It would be thought-provoking to investigate the impact of SI and ICT infrastructure on income inequality with the moderating role of e-governance.

## Data availability statement

Data will be made available on request

## CRediT authorship contribution statement

**Sonia Bibi:** Writing – review & editing, Writing – original draft, Methodology, Formal analysis, Data curation. **Hassan Zada:** Writing – review & editing, Supervision, Investigation. **Tahira Awan:** Validation, Supervision, Methodology, Investigation, Formal analysis, Conceptualization. **Wing-Keung Wong:** Writing – review & editing, Validation, Supervision, Resources. **Naveed Khan:** Writing – review & editing, Validation, Investigation.

## Declaration of competing interest

The authors declare that they have no known competing financial interests or personal relationships that could have appeared to influence the work reported in this paper.

## Figures and Tables

**Table A1:** Variable description

Variable	Symbol	Proxy	Source
Social Inclusion	SI	policies and institutions for environmental sustainability, building human resources, equity of public resource use, social protection and labor, and Gender equality,	Country policy and institutional access
ICT Infrastructure	ICT	Wireless broadband subscriptions, mobile subscribers, fixed broadband subscriptions, and Estimated internet users, each per 100 inhabitants.	ITU
Governance	GOV	Rule of law, control of corruption, government effectiveness, political stability, regulatory quality, and voice and accountability	World governance indicators
Age dependency ratio	ADR	Ratio of dependents-people (younger than 15 or older than 64 age) to working age population (15–64 age).	World Bank
Inflation	INF	GDP deflator	World Bank
Market Size	POP	Total population	World Bank
Urbanization	UPOP	Percentage of urban population to total population	World Bank

**Table A2.b:** List of Countries

Med-High FI	Low FI
Armenia, Bangladesh, Bosnia and Herzegovina, Cabo Verde, Georgia, Honduras, India, Mauritania, Moldova, Mongolia, Vietnam, Uzbekistan	Afghanistan, Angola, Benin, Bolivia, Burkina Faso, Burundi, Cambodia, Cameroon, Chad, Côte d'Ivoire, Djibouti, Ghana, Guinea, Guyana, Kenya, Kyrgyz Rep., Lao, Lesotho, Madagascar, Malawi, Mali, Mozambique, Nepal, Nicaragua, Niger, Nigeria, Pakistan, Rwanda, Senegal, Tajikistan, Togo, Uganda, Zambia, Zimbabwe

**Table A3.a:** Total Variation Explained/ Correlation

Total variance explained			
Availability			
Component	Eigenvalue	Proportion	Cumulative
Comp1	1.76215	0.8811	0.8811
Comp2	0.237849	0.1189	1
Accessibility			
Component	Eigenvalue	Proportion	Cumulative
Comp1	1.81606	0.908	0.908
Comp2	0.183941	0.092	1
Usage			
Component	Eigenvalue	Proportion	Cumulative
Comp1	1.93949	0.9697	0.9697
Comp2	0.060512	0.0303	1
FI			
Component	Eigenvalue	Proportion	Cumulative
Comp1	1.57462	0.5249	0.5249
Comp2	0.796875	0.2656	0.7905
Comp3	0.6285	0.2095	1
ICT Infrastructure			
Component	Eigenvalue	Proportion	Cumulative
Comp1	2.81565	0.7039	0.7039
Comp2	0.60795	0.152	0.8559
Comp3	0.385835	0.0965	0.9524
Comp4	0.190561	0.0476	1
Governance			
Component	Eigenvalue	Proportion	Cumulative
Comp1	4.1506	0.6918	0.6918
Comp2	0.762397	0.1271	0.8188
Comp3	0.480938	0.0802	0.899
Comp4	0.275543	0.0459	0.9449
Comp5	0.203402	0.0339	0.9788
Comp6	0.127123	0.0212	1

**Table A3.b:** Component Loadings

Variable	PC1	PC2	PC3	PC4	PC5	PC6
Availability						
BB	0.7071	0.7071				
ATM	0.7071	−0.7071				
Accessibility						
NBB	0.7071	0.7071				
NATM	0.7071	−0.7071				
Usage						
OD	0.7071	0.7071				
OL	0.7071	−0.7071				
Availability	0.588	−0.5137	0.6249			
Accessibility	0.6204	−0.2094	−0.7558			
usage	0.5191	0.8321	0.1955			
FB	0.4796	−0.6828	0.2367	0.4977		
AM	0.5	0.3997	−0.666	0.3831		
MC	0.4736	0.5577	0.6814	−0.0154		
IU	0.5437	−0.251	−0.19	−0.778		
CC	0.4434	−0.0258	−0.0297	−0.6806	−0.4003	0.4223
GE	0.4327	0.0642	−0.4561	−0.0316	0.7531	0.1804
PS	0.2947	0.8671	0.3319	0.212	−0.0484	0.0626
REG	0.4238	−0.2031	−0.3567	0.6544	−0.4497	0.1461
LAW	0.4639	−0.0471	−0.0193	−0.1852	−0.0756	−0.8615
VA	0.3667	−0.4471	0.7439	0.1679	0.2497	0.1472

**Table A4:** List of Abbreviation

Abbreviation	Definition
BB	Bank branches/100,000 adults
ATM	ATM/100,000 adults
NBB	Bank branches/1,000 ${km}^{2}$
NATM	ATM/1,000 ${km}^{2}$
OD	Outstanding deposits % of GDP
OL	Outstanding Loans % of GDP
FB	Fixed broadband subscriptions/100 persons
AM	Active mobile broadband subscriptions/100 persons
MC	Mobile subscribers/100 persons
IU	Internet users/100 persons
CC	corruption control
GE	Government effectiveness
PS	Political stability
REG	Regulatory quality
LAW	Rule of law
VA	Voice and accountability

## Appendix

### Table A2.a. List of Countries

Afghanistan, Angola, Armenia, Bangladesh, Benin, Bolivia, Bosnia and Herzegovina, Burkina Faso, Burundi, Cabo Verde, Cambodia, Cameroon, Chad, Côte d'Ivoire, Djibouti, Georgia, Ghana, Guinea, Guyana, Honduras, India, Kenya, Kyrgyz Rep, Lao, Lesotho, Madagascar, Malawi, Mali, Mauritania, Moldova, Mongolia, Mozambique, Nepal, Nicaragua, Niger, Nigeria, Pakistan, Rwanda, Senegal, Tajikistan, Togo, Uganda, Uzbekistan, Vietnam, Zambia, Zimbabwe.
